# Editorial: Trends and perspectives for the use of crop wild relatives in crop breeding, volume II

**DOI:** 10.3389/fpls.2026.1995090

**Published:** 2026-09-22

**Authors:** Rodomiro Ortiz, Mahesh Rao, Filippo Bassi

**Affiliations:** 1 Department of Plant Breeding, Swedish University of Agricultural Sciences, Alnarp, Sweden; 2 Indian Council for Agricultural Research – National Institute for Plant Biotechnology, Pusa, New Delhi, India; 3 International Center for Agricultural Research in the Dry Areas, Rabat, Morocco

**Keywords:** genotyping, interspecific hybridization, introgression, phenotyping, wild species

Crop wild relatives (CWRs) are crucial assets for agrobiodiversity, sustainable agriculture, and food security, offering vital genetic traits such as pest resistance, resource efficiency, and extreme weather adaptability (Dwivedi et al., 2007). Domestication bottlenecks and the heavy reliance on elite breeding lines have, however, caused severe genetic erosion in modern crops, limiting their adaptive potential. Although CWRs and their associated microbiota hold vast potential to counter these vulnerabilities (Ortiz et al., 2024), they face severe environmental threats and underutilization due to biological barriers and knowledge gaps (Bassi et al., 2024). Overcoming these constraints through advanced genomic resources, phenotypic characterization, and interspecific hybridization is essential to harnessing wild and landrace diversity for climate-resilient crop improvement, as noted in the 10 articles collected in this Research Topic in *Frontiers in Plant Science*.

Avasiloaiei et al. indicated that CWRs and landraces are vital genetic reservoirs for enhancing legume crops against escalating abiotic stresses and biotic pressures. The authors explained that domestication significantly narrowed the cultivated legumes’ genetic base, thus limiting their yield stability under climate change. CWRs harbor crucial alleles for physiological resilience, stress signaling, and symbiotic nitrogen fixation in major species such as bean (*Phaseolus vulgaris*), chickpea (*Cicer arietinum*), and soybean (*Glycine max*). Integrating these wild gene pools with landraces via modern breeding innovations, such as high-throughput phenotyping, multi-omics, and gene editing, allows researchers to capture complex multigenic traits, minimize linkage drag, and accelerate the development of climate-resilient cultivars to ensure sustainable global food security.

*Artemisia argyi* –a traditional Chinese medicine plant– faces germplasm and cytological research hurdles due to its complex genomic structures. To overcome this, Li et al. used the reference genome to develop 20 repetitive sequence oligonucleotide probes configured into two multiplex cocktails for non-denaturing fluorescence *in situ* hybridization. Combining these with 45S rDNA markers and electronic localization established a high-resolution, genome-map-based karyotype for the cultivar ‘Qicun Xiang Ai’. This framework enabled chromosome identification across diverse cultivars, revealing that *A. argyi* is a disomic tetraploid (4*x*) with a basic chromosome number (*x*) of 17. Additionally, chromosomal doubling successfully generated a novel octoploid (2*n* = 8*x*) germplasm that exhibited significantly enhanced length of moxa – a pliable substance produced by pulverizing dried mugwort foliage, traditionally utilized in East Asian healing practices– and leaf-area content.

*Beta vulgaris* ssp. *maritima* (sea beet), the wild ancestor of cultivated sugar beet, serves as a vital reservoir of adaptive genetic diversity. Research into sea beet populations across Europe and North Africa reveals extensive morphological variation, such as prostrate coastal growth habits versus water-efficient inland forms, alongside a distinct Atlantic-Mediterranean genetic divide (Bertram and Frisch). While Mediterranean and southern/eastern populations exhibit high genetic diversity and early bolting, northern populations show genetic homogeneity due to founder effects, with phenotypic variation frequently outpacing genetic differentiation. The authors established an essential resource for future sea beet conservation and sugar beet breeding. Furthermore, to address the limited genetic variation within elite sugar beet breeding pools, Bertram et al. analyzed 1,363 genotypes across Northern Atlantic populations from Denmark, France, and Ireland using 16,201 single nucleotide polymorphisms (SNPs). Their investigation revealed stark structural differences: the Danish cohort exhibited low genetic variation and minimal structure, the French group showed intermediate levels, and the Irish population displayed the highest diversity alongside pronounced population stratification. Consequently, while Irish wild relatives hold the greatest potential for direct trait discovery via association mapping, successfully leveraging this reservoir requires effectively managing complex population structures during statistical analyses.

Verma et al examined 42 local accessions using 25 morphological traits and 17 informative simple sequence repeat (SSR) markers to evaluate the genetic architecture of Deenanath grass (*Pennisetum pedicellatum*), a vital tropical forage crop. *D*^2^ cluster analysis grouped the genotypes into six distinct clusters, revealing substantial morphological and molecular variation, with molecular similarity coefficients ranging from 0.43 to 0.87. Population structure analysis further delineated the germplasm into wild and cultivated subpopulations. Because of their superior combining potential and high diversity indices, specific accessions such as RDC-20, RDC-21, and RDC-6 were identified as promising parental sources to drive future breeding programs targeting enhanced productivity, environmental adaptability, and stress resilience.

Wild rice species provide a crucial genetic reservoir for breeding elite cultivars, but leveraging CC-genome wild species within the genus *Oryza* has been hindered by a lack of efficient, genome-specific molecular tools. To bridge this gap, Lopez et al. developed a comprehensive set of 182 genome-wide polymorphic insertion-deletion (InDel) markers via sequence alignments between *O. officinalis* (CC-genome) and cultivated rice (*O. sativa*, AA-genome). Validated across diverse wild accessions and cultivars using polymerase chain reaction (PCR)-agarose gel analysis, 172 of these markers exhibited high polymorphism. Furthermore, specific subsets successfully differentiated closely related CC-genome species and accurately detected wild introgressions in wide hybridization progenies, providing essential genomic tools for advanced rice improvement.

Peanut or groundnut (*Arachis hypogaea*) suffers from a narrow genetic base, making it vulnerable to various pests, pathogens, and abiotic stresses. Although wild *Arachis* species offer high resistance, a ploidy barrier complicates direct interspecific hybridization for peanut breeders. To overcome this challenge, Barnes et al. developed a structured introgression line (IL) population comprising 32 lines derived from the wild species *A. stenosperma* and *A. batizocoi*. This population provides broad genome coverage, representing significant polymorphic marker percentages and wide phenotypic diversity—including host plant resistance to pathogens and varying plant height. To be freely released into the public domain, this resource enables peanut breeders to harness wild genetic value without undertaking laborious hybridizations.

*Leymus mollis* acts as a valuable tertiary gene pool for wheat improvement, providing crucial host plant resistance to pathogens and stress tolerance. Du et al. characterized two novel wheat-*L. mollis* translocation lines, namely, M892 and M956, to exploit this potential. Phenotypic testing demonstrated that M956 exhibits high resistance to both stripe rust and *Fusarium* head blight, whereas M892 is susceptible. However, both lines show improved grain weight, with dwarfism in M892. Cytogenetic characterization identified M892 as an insertional translocation line and M956 as a terminal translocation line. Furthermore, genetic mapping and transcriptomics identified 47 candidate resistance genes within a critical 98-Mb region on chromosome 4NsL, supported by eight new molecular markers for marker-aided breeding.

Sweet potato (*Ipomoea batatas*) is an important global food crop severely threatened by the sweet potato weevil (*Cylas formicarius*), which causes significant yield loss in these tuberous roots. Morales et al. evaluated 731 diverse accessions from a global germplasm collection using field and laboratory bioassays to identify resistance markers. The results revealed that 80% of the accessions were highly susceptible, with only 6.5% showing resistance. Resistance correlated most strongly with deep storage root positioning, which acts as a physical escape mechanism. The authors also indicated that discovering an exceptionally resistant genotype with shallow roots highlights a unique biochemical defense mechanism, paving the way for targeted breeding programs to enhance plant health management in sweet potato.

In a comprehensive study, Xue et al. evaluated the phenotypic variability of 1,558 accessions of foxtail millet (*Setaria italica*), which is an important specialty mixed grain crop originating in China. Foxtail millet possesses significant potential for agricultural diversification and food security. Xue et al. investigated 25 distinct traits comprising 11 quantitative and 14 qualitative descriptors using physical, chemical, and digital image analysis techniques. Their findings revealed significant phenotypic variation, with coefficients of variation for quantitative characteristics ranging broadly from 3.9% to 28.4%, thus offering essential baseline data for future foxtail millet breeding.

In summary, CWRs and landraces serve as key genetic reservoirs for enhancing agrobiodiversity, resilience, and global food security. This Research Topic in *Frontiers in Plant Science* shows, indeed, that diverse innovations exist across various gene pools. High-resolution karyotyping, diversity assessments, and new genomic tools are advancing the use of CWRs and landraces for the improvement of the cultigen pool of various crops, thus accelerating the development of climate-resilient cultivars.
